# Three-dimensional structure and cytokine distribution of platelet-rich fibrin

**DOI:** 10.6061/clinics/2017(02)09

**Published:** 2017-02

**Authors:** Meng-Yi Bai, Ching-Wei Wang, Jyun-Yi Wang, Ming-Fang Lin, Wing P Chan

**Affiliations:** I Graduate Institute of Biomedical Engineering, National Taiwan University of Science and Technology, Taipei 10607, Taiwan; II Department of Radiology, Wan Fang Hospital, Taipei Medical University, Taipei 116, Taiwan; III Department of Radiology, School of Medicine, College of Medicine, Taipei Medical University, Taipei 110, Taiwan; IV Adjunct appointment to the Department of Biomedical Engineering, National Defense Medical Center, Taipei 116, Taiwan

**Keywords:** Biomaterial, Cytokine, Platelet-Rich Fibrin (PRF)

## Abstract

**OBJECTIVES:**

Previous reports have revealed that several cytokines (including platelet-derived growth factor-BB, transforming growth factors-β1 and insulin-like growth factor-1) can enhance the rate of bone formation and synthesis of extracellular matrix in orthopaedics or periodontology. This study aimed to determine the concentration of cytokines within platelet-rich fibrin microstructures and investigate whether there are differences in the different portions of platelet-rich fibrin, which has implications for proper clinical use of platelet-rich fibrin gel.

**METHODS:**

Whole blood was obtained from six New Zealand rabbits (male, 7 to 39 weeks old, weight 2.7-4 kg); it was then centrifuged for preparation of platelet-rich fibrin gels and harvest of plasma. The resultant platelet-rich fibrin gels were used for cytokine determination, histological analyses and scanning electron microscopy. All plasmas obtained were subject to the same cytokine determination assays for the purpose of comparison.

**RESULTS:**

Cytokines platelet-derived growth factor-BB and transforming growth factor-β1 formed concentration gradients from high at the red blood cell end of the platelet-rich fibrin gel (*p*=1.88×10^-5^) to low at the plasma end (*p*=0.19). Insulin-like growth factor-1 concentrations were similar at the red blood cell and plasma ends. The porosities of the platelet-rich fibrin samples taken in sequence from the red blood cell end to the plasma end were 6.5% ± 4.9%, 24.8% ± 7.5%, 30.3% ± 8.5%, 41.4% ± 12.3%, and 40.3% ± 11.7%, respectively, showing a gradual decrease in the compactness of the platelet-rich fibrin network.

**CONCLUSION:**

Cytokine concentrations are positively associated with platelet-rich fibrin microstructure and portion in a rabbit model. As platelet-rich fibrin is the main entity currently used in regenerative medicine, assessing cytokine concentration and the most valuable portion of PRF gels is essential and recommended to all physicians.

## INTRODUCTION

Platelet-rich fibrin (PRF) is a second generation platelet-containing biomaterial with potential applications in wound healing. Unlike the first generation of platelet-rich products, such as platelet-rich plasma (PRP), PRF does not require the addition of an anticoagulant (such as ethylenediaminetetraacetic acid [EDTA]) during the initial drawing of blood, nor calcium chloride nor bovine thrombin to induce polymerization 1,2. PRF traps platelets and platelet cytokines in a fibrin gel. Its multitude of cytokines at concentrations significantly higher than the baseline blood levels stimulates autologous regeneration 3. Articular cartilage repair 4-6, regeneration after oral and maxillofacial surgery 7-9, and wound healing 10-12, all depend on the presence of cytokines that stimulate the migration and proliferation of cells within the affected part of the body. Quantification of the platelet cytokines in PRF is essential to evaluating its efficacy as a biomaterial for regenerative medicine.

The ultrastructure and fibre thickness of rabbit PRF are similar to those of human fibrin 13. Thus, in a previous study, to explore the potential use of PRF gel in regenerative medicine, we used an experimental rabbit model to investigate the regeneration of articular cartilage defects in the knee; this model consisted of autologous implantation of PRF and minced cartilage fragments into injured knees 4. Although the study indicated that the observed healing might be due to the release of cytokines from the autologous PRF, no qualitative or quantitative evidence of platelet cytokines in the PRF was presented, limiting the preclinical elucidation of the study and its translation to clinical treatment of articular cartilage injury.

Dohan and coworkers 14 quantified the cytokine concentrations in the three layers formed by centrifugation of blood in a collection tube (red blood cells [RBC], PRF, and plasma), but they considered the middle layer (i.e., the PRF gel) to be the only potential cytokine source for clinical practice and therefore focused on measuring the concentration of platelet cytokines in PRF gels. In another study, it was shown that fibrin architecture and leukocyte content influence cytokine release from four different families of platelet concentrates, not including PRF 15,16. The abovementioned findings inspired us to ask whether cytokines are concentrated and evenly distributed in PRF gel, as this is the main entity currently used in regenerative medicine, for example, in dentistry 17,18.

In this study, we focused on PRF gel because this is the only autologous family of platelet concentrates without the addition of foreign reagent and able to reserve and release cytokines in a controlled manner 19. Three platelet cytokines (platelet-derived growth factor-BB: PDGF-BB, transforming growth factor-beta 1: TGF-β1 and insulin-like growth factor 1: IGF-1) were chosen for analyses because their biological activities during initial healing are well understood 20. Although research on human cytokines quantification has been widely reported 21-25, we approached the issue differently in this study by quantifying cytokines obtained from districted PRF, not global PRF, in rabbits. It is because of the unexpected discovery of an uneven distribution of cytokines in our previous study to elucidate the potential use of PRF gel in regenerated articular cartilage defects in rabbits’ knees. We therefore raised two questions: first whether these cytokines are indeed concentrated in the PRF gel rather than in plasma, and second, if so, whether they are evenly distributed in the fibrin network of the PRF gel. These questions led us to determine the cytokine content of PRF and its relationship with the three-dimensional fibrin network structure.

## MATERIALS AND METHODS

### Preparation of PRF gel and matrix

Six New Zealand rabbits (male, 7 to 39 weeks old, weight 2.7-4 kg) were used for the preparation of PRF gels. The animal breeding practices and animal use protocol were approved by the Institutional Animal Care and Use Committee (IACUC) at Taipei Medical University (certificate no. LAC-101-0300). The rabbits were maintained according to the regulations of the IACUC at our institution, where they were housed. All experiments were performed in accordance with the replace, reduce, and refine principles outlined in the IACUC guidelines. The number of animals was the minimum required to obtain statistically valid results. The sample size per group was calculated based on an estimated between-group difference of 0.5, assuming a two-sided confidence interval of 95% and a variance of 0.5.

**Figure 1:**
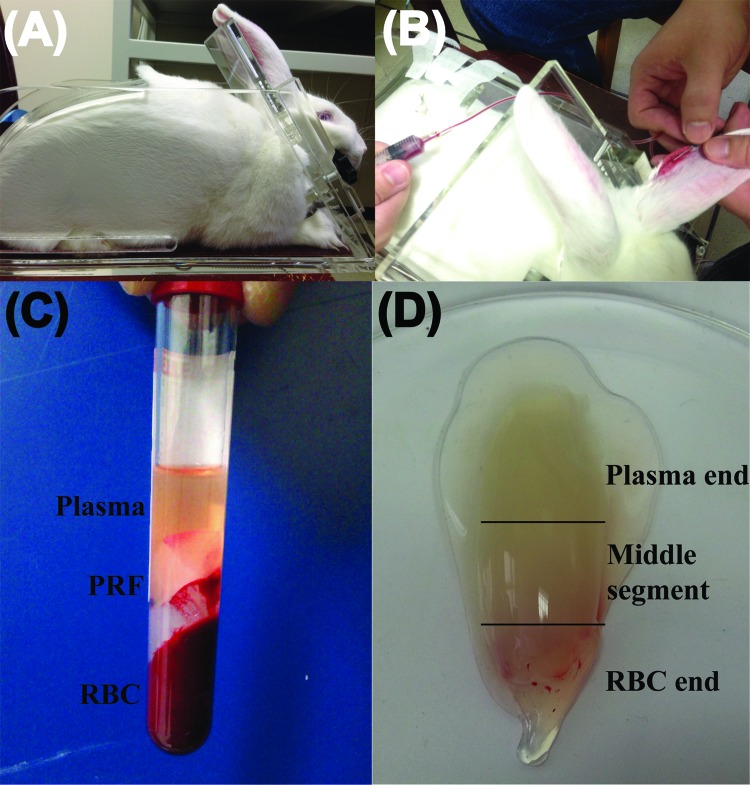
Preparation of platelet-rich fibrin (PRF) gel using blood from New Zealand rabbits. (A) A rabbit is placed in a restraining frame. (B) Blood is drawn from an ear vein. (C) A 5-mL blood sample is separated into three layers by centrifugation at 3000 rpm for 10 min. (D) A PRF gel forms between the plasma layer (top) and the red blood cell (RBC) layer (bottom).

PRF was prepared using the protocol that was described for the first time internationally by Dohan et al. in 2006 26 with slight modifications to improve reproducibility. Because only rabbits above 3 kg body-weight have large enough ear veins for venipuncture, these larger rabbits were placed in a restraining device to facilitate the drawing of blood (Figure 1a). In addition, xylene (ACS reagent, J. T. Baker, Phillipsburg, NJ, USA) was applied to the skin over the ear vein to dilate the vessel and assist in locating the vein. Five millilitres of blood were drawn from the ear vein (Figure 1b) with a no. 21 gauge butterfly needle, immediately transferred to an 8-mL sterile plastic tube (without anticoagulant, Vacuette® tube, 2 mL Z no additive, item no. 455071), and centrifuged at 3000 rpm for 10 min in a DSC-200A-2 tabletop centrifuge (Digisystem, Laboratory Instruments Inc., Taipei, Taiwan). It is noteworthy that only the venous blood could successfully produce the PRF gel after it was subject to centrifugation (Figure 1b-c, confirmation that blood was drawn from the rabbit auricular vein, not from the central ear artery). A pair of forceps was used to remove the middle layer (the whole PRF gel, Figure 1d) from the top layer (plasma) and bottom layer (RBC). The plasma was then harvested and stored in an Eppendorf tube (Avant® microcentrifuge tube, 1.7 mL, certified free of RNase/DNase, human DNA, Pyrogen, PCR inhibitors) for the following analyses. A representative PRF gel prepared from centrifugation of 5 mL of whole blood is shown in Figure 1d. Before placing the PRF sample in the freezer, the surface adsorbing RBC layer fluid had to be completely drained away to avoid interference with the cytokine determination assays. The PRF (a pale yellow elastic gel-like strip) was frozen at −20°C for 30 min and then transferred to another freezer at −80°C for storage until the cytokines were quantified. This gradual freezing protocol mimics the protocol for freezing cells to avoid denaturation of cytokine proteins as cytokine levels can be decreased by improper handling and storage of samples 27.

Each of the six PRF samples was divided into three segments for cytokine quantification. Normally, the pointed tip on the RBC end was trimmed off, as RBC contamination strongly influenced cytokine determination using the spectrometer. Therefore, cytokines from the RBC layer were excluded. Then, cytokine activation was conducted in 4-(2-hydroxyethyl)-1-piperazineethanesulfonic acid (HEPES, ≥99.5%, Sigma-Aldrich, St Louis, MO, USA, pH 7.2-7.6). Cytokine concentration was measured using sandwich-type ELISA kits (nos. MBS165883, MBS165545, and MBS704445, MyBiosource, Inc., San Diego, CA, USA) according to the manufacturer’s instructions. After the exudate was removed from the defrosted PRF gel, the remaining white PRF matrix was fixed in 10% formalin (Sigma-Aldrich) for 22 h and dehydrated in a graded ethanol series (70% to 100%) for the following preparation of histological specimens.

### Histological and scanning electron microscopic analysis

**Figure 2:**
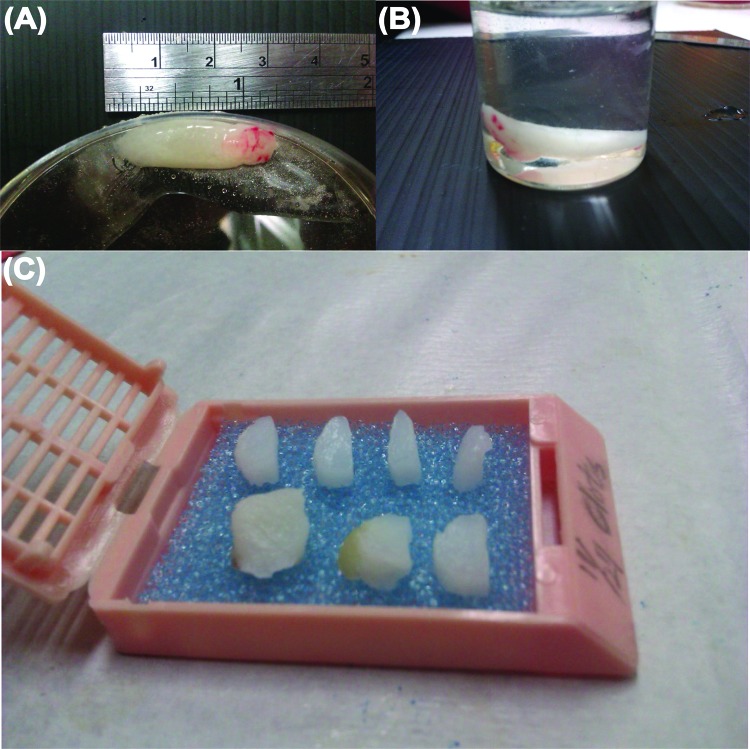
Photographs showing (A) a defrosted PRF gel, (B) a PRF gel fixed in 10% formalin for 22 h, and (C) the fixed PRF gel embedded in paraffin, divided into 7 segments and sent for paraffin sectioning. The two ends of the PRF gel were discarded due to distortion.

The PRF sample was fixed, dehydrated and placed in a tissue cassette and embedded in paraffin wax. Infiltration of paraffin into the porous tissue maintains the intrinsic morphology of the tissue and solidifies the sample to allow fine sectioning. The sample was divided into seven segments (Figure 2a-c), then cut into 3–5 μm sections for histological analyses. The RBC end and plasma end of the PRF were similar and easily confused during this preparation of histological samples. Sample orientation errors were avoided by labelling. The tissue sections were mounted on slides, stained with haematoxylin-eosin or Masson’s trichrome stain, viewed and photographed under an optical microscope 28. The compactness of the fibrin network (i.e., porosity, [n]) was measured directly by scanning electron microscopy (SEM) using ImageJ software (NIH, Bethesda, MD, USA). Porosity [n]=A_p_/A_t_ × %, where A_p_ denotes the measured total area of the pores in each SEM image and A_t_ is the total area of an SEM image.

All protocols of histological and SEM analyses for the animal rabbit model were developed for this PRF sample and were slightly modified from the typical tissue preparation method for light microscopy. Optical microscopy and SEM were used in a complementary fashion, as they have different levels of resolution for inspecting the fibrin microstructure (the resolution of the optical microscope falls within the μm range and that of the SEM can be in nm). To prevent charge accumulation and damage to tissue sections during SEM examination, approximately 80 Å of platinum was deposited on the tissue sections using a sputter coater. SEM images were acquired using a field-emission scanning electron microscope (FESEM, Model JSM-5600, JEOL, Tokyo, Japan) operated at an accelerating voltage of 15 kV. Serial images were aligned sequentially in three-dimensional space and visualized three-dimensionally with Fiji and TrakEM2 software 29,30. In a typical procedure, serial slides can be manually aligned by setting up a number of pairs of corresponding control points to the same (x, y) location for consecutive images z_i_ and z_i+1_, and the pairs of images and paired-sets of control points are then given to semi-automatic software for image alignment. Fully automatic registration of biological images is then possible as demonstrated by the software - TrakEM2 29,30. The average diameters of the fibrin fibres were obtained by averaging diameters directly measured in the SEM images of ∼30 samples.

### Statistical analysis

The concentrations of cytokines from six rabbits are expressed as the mean ± SD for each type of cytokine. Student’s *t*-test was used to compare results for PRF and plasma (the control). *P*<0.05 was considered statistically significant. Excel® (Microsoft, Seattle, WA, USA) was used for statistical analysis.

## RESULTS

### Cytokine measurement

**Figure 3:**
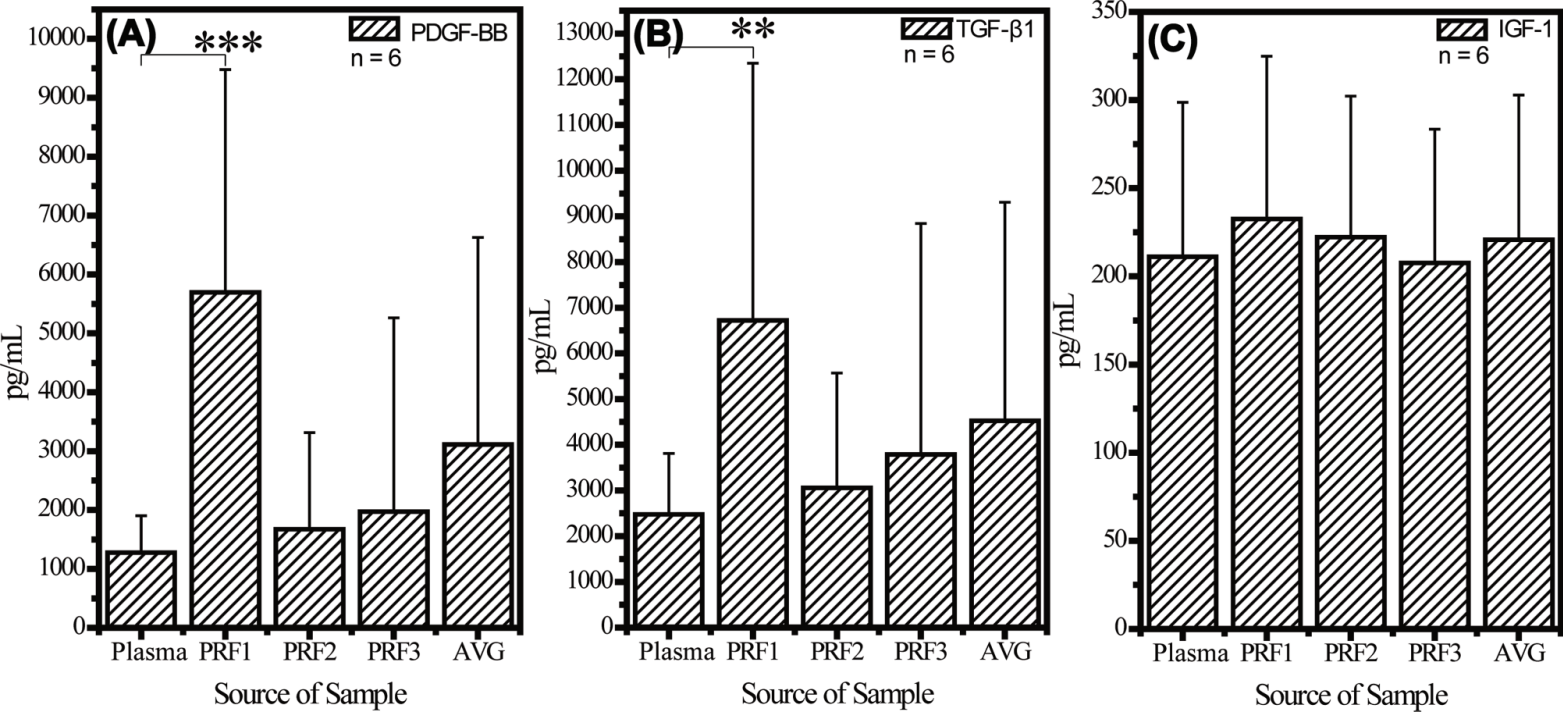
Cytokine quantification assay of (A) PDGF-BB, (B) TGF-β1, and (C) IGF-1 in PRF gel. All values are expressed as the mean ± SD. The RBC end, middle segment, and plasma end of the PRF clot are labelled PRF1, PRF2, and PRF3, respectively. AVG is the average of the PRF1, PRF2 and PRF3 values. Concentrations of PDGF-BB and TGF-β1 in PRF1 were always two to five times that in PRF3, but the concentration of IGF-1 in PRF1 did not differ significantly from that in PRF3. *p* value <0.01 is labelled **, and *p* value <0.001 is labelled ***. All statistical results are with respect to the group of plasma.

Figure 3 shows data obtained from all six rabbits. PDGF-BB and TGF-β1 formed quasi-gradients of concentration from high at the RBC end (PRF1, *p*=1.88×10^-5^ compared with the level in plasma) to low at the plasma end (PRF3, *p*=0.19). IGF-1 concentrations were similar at the RBC and plasma ends (no statistical significance was observed).

### Histological and SEM observations

**Figure 4:**
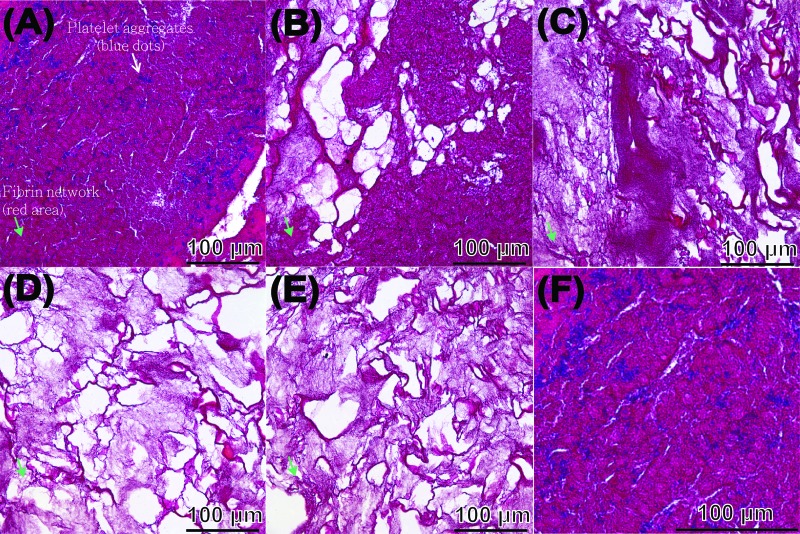
The five segments obtained from the PRF sample shown in Figure 2 were used for histological analysis, and sections were stained with Masson’s trichrome. Sections from the RBC end (A), middle segment (B-D), and plasma end (E) of the PRF show a decrease in compactness and increase in porosity as the view of the PRF gel shifts from the RBC end to the plasma end. A micrograph (F) taken at high magnification from (A), revealing finer structural details.

**Figure 5:**
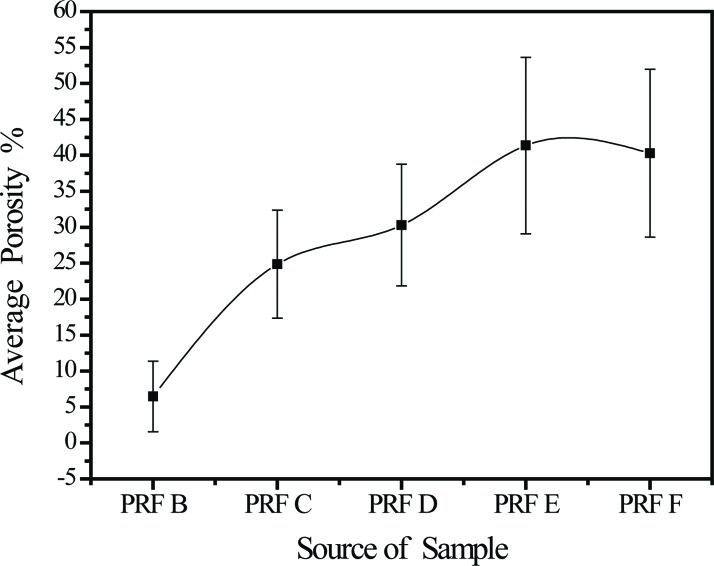
Statistics concerning the porosity of the PRF specimens shown in Figure 2 (for interpretation of the sample labelling, please refer to Figure 6a).

**Figure 6:**
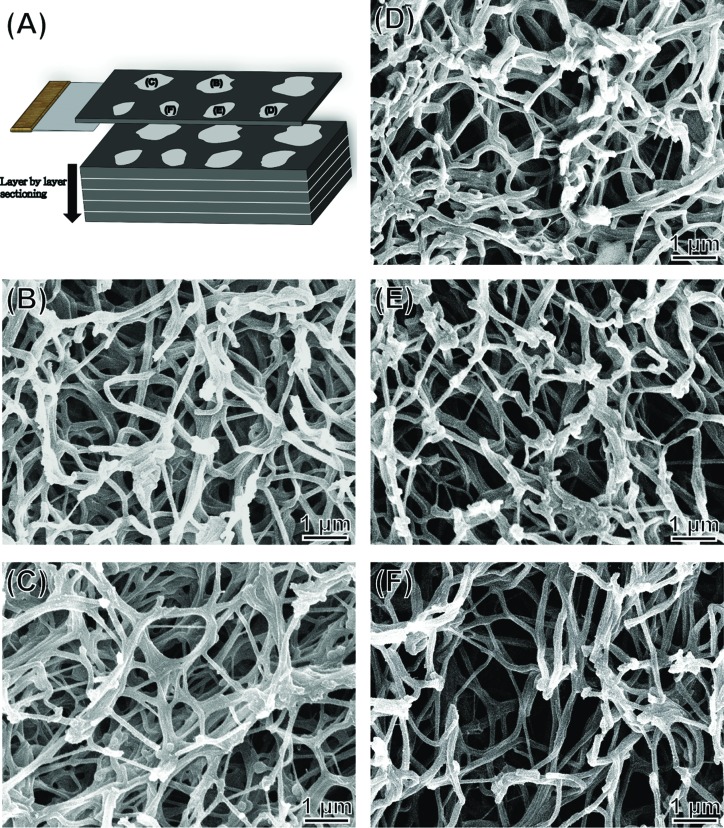
(A) Diagrammatic view of a sectioned PRF gel and (B–F) SEM images of the series of PRF sections shown in Figure 2. Labels in (A) indicate the five segments of the PRF sample observed in the SEM micrographs.

Compactness and porosity in the PRF tended to be non-uniform (Figure 4). The fibrin network (see the red area indicated by the green arrowheads) closest to the RBC layer had particularly small pores and an ultra-compact structure with many incorporated platelets (see the blue spots indicated by the white arrowheads, the PRF section observed in Figure 4a corresponds to PRF1 shown in Figure 3) and that closest to the plasma layer had larger pores and a less compact structure (the PRF section observed in Figure 4e corresponds to PRF3 shown in Figure 3, the remaining images, i.e., Figure 4b-d, correspond to PRF2 shown in Figure 3). The porosities of these PRF samples taken in sequence from the RBC end to the plasma end were 6.5% ± 4.9%, 24.8% ± 7.5%, 30.3% ± 8.5%, 41.4% ± 12.3%, and 40.3% ± 11.7% (Figure 5, as illustration of sample sites shown in Figure 6a), indicating a gradual decrease in the compactness of the PRF network.

**Figure 7:**
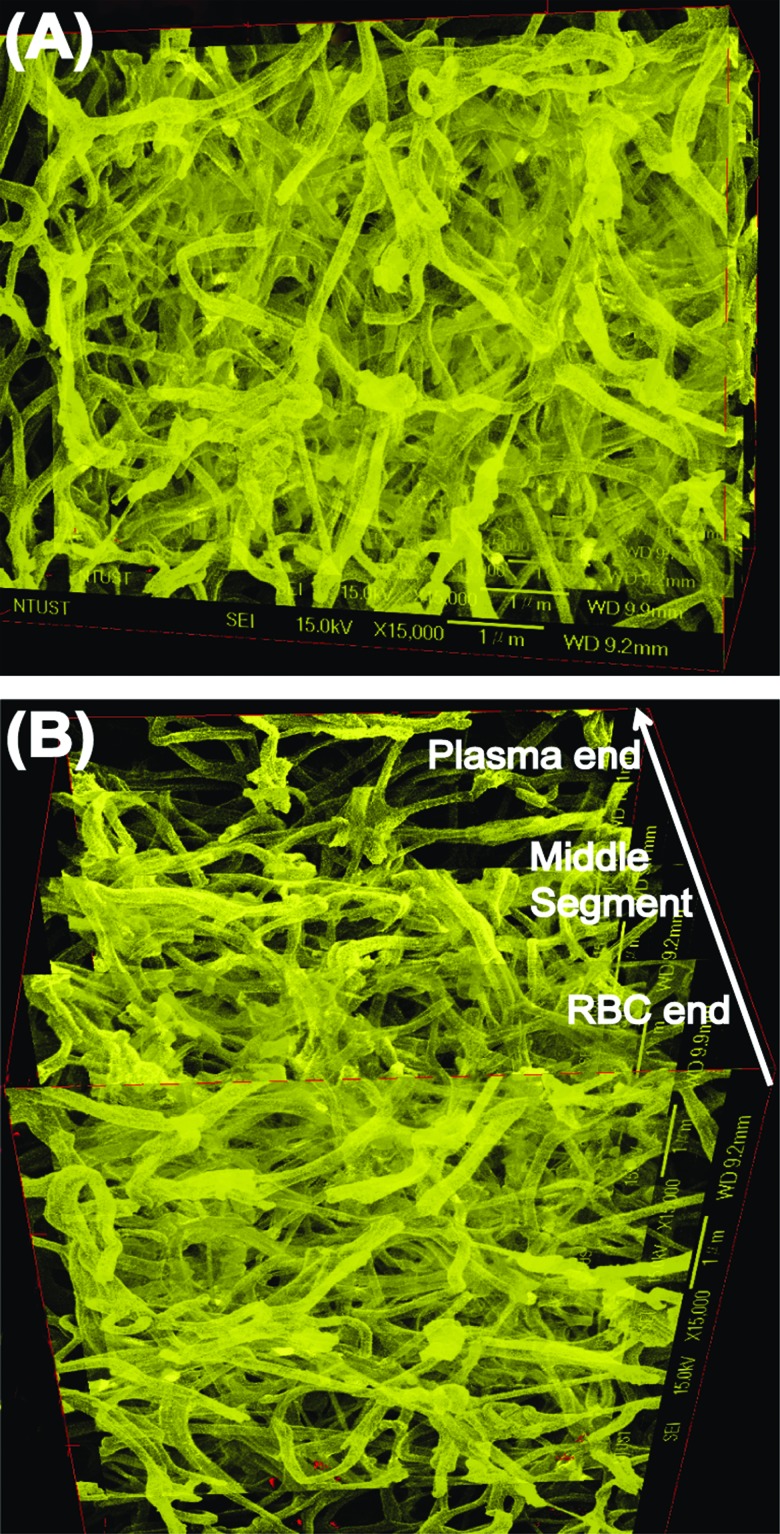
Three-dimensional image constructed from the SEM images shown in Figure 6. (A) Top view showing the PRF gel from the RBC end, and (B) side view showing the cross-section of the PRF gel. An arrow indicates the direction of gradually decreasing compactness and increasing porosity of the PRF microstructure from the RBC end to the plasma end.

SEM images showed that the fibrin fibres in the five segments of a representative PRF sample (images taken sequentially from the RBC end to the plasma end; Figure 6a) had average diameters of 116 ± 37, 116 ± 39, 123 ± 37, 124 ±38, and 140 ± 39 nm, respectively. In addition, because robust and fully automatic 3D registration of serial-section microscopic images is critical for detailed anatomical reconstruction of large biological specimens, such as the case of PRF histology shown above. This construction image can help with providing more information about the essence of platelet-rich fibrin (ePRF) to gain new structural insights. On reconstructing the two-dimensional SEM images and viewing from above, the three-dimensional reconstruction image of the fibrin matrix was dense and similar in appearance to medical gauze (Figure 7a). From the side-view, the compactness decreased and porosity increased along the longitudinal axis of the PRF sample from the RBC end to the plasma end (Figure 7b).

## DISCUSSION

This study confirms that platelet cytokines are indeed trapped in PRF gel but contradicts the hypothesis of Mosesson et al. 31,32 that the trapped platelet cytokines are evenly distributed throughout the fibrin network. The formation of a three-dimensional fibrin network resulted in the incorporation of cytokines into its mesh architecture. We propose that fibrinogen and thrombin concentrations are higher at the RBC end of a PRF sample, possibly because of the force of centrifugation. Our results agree with those of Ryan and Wang 33,34, who demonstrated that a higher concentration of thrombin results in the generation of fibrin with a smaller diameter and that these smaller fibrin fibres tend to pack more tightly, such that the porosity is lower. Our results provide clinically useful information that could support the development of improved therapeutic interventions, as the potential curative effect of PRF arises mainly from its high concentration of incorporated cytokines.

Images of histologically stained PRF show fibrin networks with varying degrees of compactness. To the best of our knowledge, the three-dimensional fibrin network of leukocyte-PRF (L-PRF) first reported by Dohan et al. in 2006 is the catalyst for the incorporation of cytokines into the mesh architecture. However, this idea is based on theoretical computer modelling of a PRF clot, not empirical evidence. Our three-dimensional representation of the PRF microstructure reconstructed from a series of two-dimensional SEM images shows a dense fibrin matrix with surface morphology similar to that of medical gauze. This tangled fibrous architecture, in contrast to the architecture of the first-generation platelet concentrate commonly known as PRP, is responsible for the slower release of growth factors and the entrapment of more platelets inside the gel 21,35. Our findings suggest that the gradient distribution of cytokines in PRF gel is strongly related to its three-dimensional structure, especially the porosity and compactness of the fibrin network. Although this structure-cytokine gradient relationship seems tied to the conditions of PRF preparation, such as types of tube for collecting blood, types of centrifuge, centrifugal force and duration of centrifugation 36, it has not been possible to establish this relationship by conducting systematic empirical investigation because of the narrow window for successful *in vivo* preparation of PRF when the L-PRF technique was applied to an animal (rabbit) model. However, *in vitro*, Ryan and Wang 33,34 have shown that adding higher concentrations of thrombin results in the generation of fibrin with a smaller diameter.

When a whole blood sample is centrifuged, a gelled membrane (the buffy coat layer) forms between the RBC layer and the plasma layer. The buffy coat contains most of the platelets, which are activated during centrifugation. The surface of these activated platelets is known to contain glycoprotein IIb/IIIa, which is a receptor for binding the soluble fibrinogen protein in fluid 37. The activated platelets also stimulate the formation of thrombin and induce the subsequent polymerisation of fibrinogen to form fibrin molecules. These adhere to one another and then become assembled into long fibrils. The formation of this fibrin gel is generally determined by the balance between the lateral aggregation of fibrinogen monomers and the rate of fibrinopeptide cleavage 38. At a high concentration of thrombin, the rate of fibrinopeptide cleavage, with simultaneous creation of many branch-points, exceeds the rate of lateral aggregation of fibrinogen monomers, giving rise to a network of thin fibres. Therefore, we propose that the concentration of fibrinogen and thrombin at the RBC end of the PRF gel is higher than that in the rest of the PRF, possibly because of the effects of density-gradient centrifugation and incorporation of platelets (Figure 4a). This provides a microenvironment conducive to the formation of a network composed of small but densely packed fibres. Our results (Figures 4–7) confirmed that the fibrin fibres at the RBC end of the PRF clot were more tightly packed (of higher density and smaller diameter) and therefore had lower porosity, so the fibres trapped more platelets, which exhibited a locking effect on the cytokines and thrombin. Conversely, the thrombin components required for conversion of fibrinogen to fibrin pass through the densely packed fibrin network from the RBC end to the plasma end of the PRF gel, generating a loosely packed fibrin network at the plasma end.

The distributions of PDGF-BB and TGF-β1, but not IGF-1, in the PRF gel were quasi-graded, and their concentrations were much higher in the PRF gel than in the plasma. Our preliminary results indicated that this effect is a combination of two factors: 1) an extrinsic factor attributed to the fibrin gel structure; and 2) an intrinsic factor attributed to the molecular structure of different cytokines. All of these cytokines were soluble and therefore should concentrate in plasma after centrifugation. However, the highest concentrations of the cytokines were at the RBC end of the gel, implying that the cytokines were stoichiometrically trapped in the PRF gel. This was a process akin to harvesting fish by casting a net into water; the process depended on the size of the mesh, which if small could remove more large granules or molecules, such as platelets, PDGF-BB (31 kDa) 39, and TGF-β1(25 kDa) 40. Because of its much lower molecular weight, IGF-1 (7 kDa) was less likely to be incorporated and retained in the fibrin matrix 41, which is consistent with the observation of Dohan et al. that IGF-1 is principally a circulating molecule 13, tending to concentrate in the upper part of the tube after centrifugation. We believe that this determination of the cytokine content of PRF produced from L-PRF technique applied to rabbit model and its relationship with the three-dimensional fibrin network structure will have a positive impact on the development of PRF products with higher clinical efficacy, such as surgical implantation of PRF in repairing articular cartilage or implanting a tooth 42 as patients desire a smaller incision, faster identification of the defect, and fewer surgeries.

In conclusion, the concentration of cytokines in a PRF gel is not uniform. The RBC end of a PRF gel contains the highest concentration of platelets and cytokines, which was thus defined as the essence of platelet-rich fibrin (ePRF). Three factors govern this distribution of cytokines: 1) the platelet distribution (which determines the concentration of thrombin); 2) the three-dimensional fibrin structure (which differs in porosity and compactness); and 3) the molecular weights of the cytokines. The structure of the PRF gel and its related cytokine distribution can inform the decision to use PRF clinically, as the efficacy of PRF is theoretically mainly dependent on the high concentrations of growth factors found within PRF compared with whole blood, which is similar to other platelet-rich products. A more extensive animal study and institutional review board (IRB)-approved human-subject study is currently comparing and extrapolating the findings of our work.

## AUTHOR CONTRIBUTIONS

Bai MY, Lin MF and Chan WP conceived and designed the experiments. Bai MY, Wang CW, Wang JY, and Lin MF performed the experiments. Bai MY and Chan WP analyzed the data. Bai MY, Wang CW, Wang JY, Lin MF, Chan WP contributed reagents/materials/analysis tools. Bai MY wrote the paper. Wang CW, Lin MF, Chan WP revised the manuscript critically for important intellectual content.
